# Microarray-Based Oncogenic Pathway Profiling in Advanced Serous Papillary Ovarian Carcinoma

**DOI:** 10.1371/journal.pone.0022469

**Published:** 2011-07-25

**Authors:** Xuan Bich Trinh, Wiebren A. A. Tjalma, Luc Y. Dirix, Peter B. Vermeulen, Dieter J. Peeters, Dimcho Bachvarov, Marie Plante, Els M. Berns, Jozien Helleman, Steven J. Van Laere, Peter A. van Dam

**Affiliations:** 1 Translational Cancer Research Unit, St Augustinus GZA Hospitals, Antwerp, Belgium; 2 Department of Gynaecological Oncology, Antwerp University Hospital, Antwerp, Belgium; 3 Cancer Research Centre, Hôpital L'Hôtel-Dieu de Québec, Centre Hospitalier Universitaire de Québec (CHUQ), Québec City, Canada; 4 Department of Medical Oncology, Erasmus MC/Josephine Nefkens Institute, Rotterdam, The Netherlands; University of Nebraska Medical Center, United States of America

## Abstract

**Introduction:**

The identification of specific targets for treatment of ovarian cancer patients remains a challenge. The objective of this study is the analysis of oncogenic pathways in ovarian cancer and their relation with clinical outcome.

**Methodology:**

A meta-analysis of 6 gene expression datasets was done for oncogenic pathway activation scores: AKT, β-Catenin, BRCA, E2F1, EGFR, ER, HER2, INFα, INFγ, MYC, p53, p63, PI3K, PR, RAS, SRC, STAT3, TNFα, and TGFβ and VEGF-A. Advanced serous papillary tumours from uniformly treated patients were selected (N = 464) to find differences independent from stage-, histology- and treatment biases. Survival and correlations with documented prognostic signatures (wound healing response signature WHR/genomic grade index GGI/invasiveness gene signature IGS) were analysed.

**Results:**

The GGI, WHR, IGS score were unexpectedly increased in chemosensitive versus chemoresistant patients. PR and RAS activation score were associated with survival outcome (p = 0.002;p = 0.004). Increased activations of β-Catenin (p = 0.0009), E2F1 (p = 0.005), PI3K (p = 0.003) and p63 (p = 0.05) were associated with more favourable clinical outcome and were consistently correlated with three prognostic gene signatures.

**Conclusions:**

Oncogenic pathway profiling of advanced serous ovarian tumours revealed that increased β-Catenin, E2F1, p63, PI3K, PR and RAS –pathway activation scores were significantly associated with favourable clinical outcome. WHR, GGI and IGS scores were unexpectedly increased in chemosensitive tumours. Earlier studies have shown that WHR, GGI and IGS are strongly associated with proliferation and that high-proliferative ovarian tumours are more chemosensitive. These findings may indicate opposite confounding of prognostic versus predictive factors when studying biomarkers in epithelial ovarian cancer.

## Introduction

Epithelial ovarian cancer (EOC) is the most important cause of mortality among gynaecological cancers. Patients with EOC often present in an advanced stage. Treatment modalities consist in general of the sequence of surgical cytoreduction and platinum-taxane based chemotherapy [1]. Although the disease is relatively sensitive to cytotoxics, relapse occurs in a majority of patients with advanced stage [1]. The emergence of resistance to conventional chemotherapeutics is an often-deadly event in the management of ovarian cancer patients. There is an urgent need for additional therapies that increase survival and/or quality of life in these patients. Recent studies of VEGF-A inhibitors have shown remarkable benefits [2]–[4]. Promising results have been reported for PARP inhibitors in ovarian cancer patients with a BRCA1 or BRCA2 mutation [5]–[7]. Individualization of therapy is necessary since epithelial ovarian cancer is a heterogeneous disease. The identification of specific targets for treatment remains a challenge.

Recent microarray technology and bioinformatics have shown the ability of analysing oncogenic cellular signalling pathways based upon gene signatures in cancers [8]–[10]. This may identify cellular processes that may be targets to develop treatment strategies. Survival can be used as a measure to quantify the biological relevance in this disease. Ideally, evaluation of survival outcome should be made in a homogenous population with a uniform treatment to avoid treatment-induced biases and uniform histology to find subtler differences independent from histology. Therefore, in the present report, patients were selected by including only patients with serous papillary histology, in advanced stages (III/IV).

Another methodology of estimating prognostic value may be the correlation with documented prognostic gene signatures that have shown to be of prognostic value in breast cancer and other types of cancer. The invasiveness gene signature (IGS) was generated using stem cell-like or tumorigenic breast cancer cells [11]. This signature has shown prognostic value in lung cancer, medulloblastoma and prostate cancer. The Wound healing response (WHR) signature, based upon genes induced by wound healing, also has shown its prognostic value in breast cancer, NSLC and bladder cancer [12]–[14]. The genomic grade index (GGI) is a signature that divides low-grade versus high-grade breast carcinomas [15]. Interestingly, using this signature, histological intermediate-grade tumours could be classified as low- or high-grade tumours with the preservation of the gene signatures' prognostic value.

The objective of this study is the analysis of oncogenic pathways in advanced serous papillary carcinoma through their relation with survival outcome and correlation with known prognostic gene signatures IGS/WHR/GGI.

## Materials and Methods

### Patient's datasets

A dataset of 285 patients (Melbourne dataset) was obtained though the Gene Expression Omnibus GEO database (GSE 9891) together with the clinical annotation data file. Only patients that had carcinomas of serous histology in advanced stages (III/IV) were included for analysis. Patients were selected that received platinum and taxane based chemotherapy. Other patients who did not receive chemotherapy or received only one agent, platinum or taxane, were also excluded. After this selection N = 165 patients were eligible for further analysis. This dataset contained gene expression data derived from the Affymetrix U133_plus2 platform, which already underwent normalisation using the Robust Multiarray Averaging (RMA) method and subsequent filtering by excluding log expression values of <7 and a variance of <0.5. After filtering there were 8,732 probe sets left that are considered informative. Progression free survival was used in further analysis [16].

A second dataset GSE3149 N = 153 (North Carolina dataset) with clinical data was also obtained from the GEO website. Here, the same criteria for patient selection were used. After selection N = 107 were further analysed. The North Carolina dataset used the same Affymetrix U133_plus2 platform. The raw data were processed in Bioconductor in R software packages. Filtering was done by selecting expressions below a threshold (log 2 of 100) that are present in at least 25% of the arrayed samples. Normalisation was done using GC-Robust Multiarray Averaging. The number of probe sets that were informative was 7,741. Overall survival data was used, as there was no progression free survival data available [8].

A third dataset (Québec dataset) were patients (N = 20) that were selected to be either chemoresistant versus chemosensitive. Here, raw microarray data based upon the Agilent platform Human 1A (v2) oligonucleotide microarray were normalised using the Lowess normalisation method. Hereafter, 16,096 genes were eligible for further analysis. Progression free survival data were used [17]. RAW gene expression data is publicly available according to MIAME guidelines through the GEO database (Accession number: GSE 28739) .

A fourth dataset (Niigata Dataset-GSE 17260) contained samples that originated from patients who met the inclusion criteria from present study. Progression free survival data were available. The authors used the Agilent Whole Human Genome Oligo Microarray platform and normalised the data using upper quartile normalisation. 28,446 genes were found to be informative [18].

A fifth and sixth dataset (Boston dataset A+B - GSE19829) were derived from a report studying BRCAness in ovarian cancer [19]. Progression free survival data was used. After selection, (N = 26) and (N = 36) patients were eligible. These datasets were RMA-normalised. 35252 and 5626 probe set ID's were informative after filtering. Gene expression data was derived from two platforms: the Affymetrix U133_plus2 platform and the Affymetrix 95UAv2.

### Oncogenic and prognostic gene signatures

The oncogenic gene signatures were derived from a recent paper by Gatza and colleagues [10] and applied similarly. Briefly, for each array-sample the pathway-specific informative genes were identified. Next a pathway score was calculated by adding up the products of the gene expression for each gene and its corresponding regression coefficient, which indicates the weight (amplitude of regression coefficient) and the effect (sign of regression coefficient) of the corresponding gene for activation of the corresponding pathway. Finally, the pathway scores were scaled using the intercept values provided in the original manuscript and standardized for comparability by median-centering and setting the standard deviation to 1. Pathways included in the analysis were AKT, β-Catenin, E2F1, EGFR, ER, HER2, INFα, INFγ, MYC, p53, p63, PI3K, PR, RAS, SRC, STAT3, TNFα, and TGFβ.

A BRCA activation score was applied using the same methodology with 60 genes, their weight and sign [19]. Prognostic gene signatures (IGS, GGI and WHR) were also applied by previously described methodology [11], [12], [15]. All gene signature activation scores were handled as a continuous variable. The same standardisation (Median = 0; SD = 1) was applied for each gene signature.

### VEGF-A activation gene signature

For the VEGF-A activation signature we used the 13 genes reported by Hu and colleagues [20]. To validate and transform this gene signature into a VEGF-A activation probability score we performed subsequent analysis using publicly available gene expression data sets on naïve and VEGF-A treated HUVEC cell lines (GSE18913 (N = 21), GSE10778 (N = 9; only the HGU133A samples were used) and GSE15464 (N = 4)). Each data set was normalised using the GC-RMA algorithm and informative genes (above log 2(100) in at least 25% of the genes) were filtered in. First, we applied a principal component analysis on the GSE18913 data set using the informative VEGF-A signatures genes only (N = 10). Significant segregation between the VEGF-A treated and naïve cell lines was investigated using class label permutation. Next, for these 10 genes, we used the regression coefficients that define the first principal component to calculate the VEGF-A activation probability score, in a similar way as described above. The score was compared between VEGF-A treated and naïve HUVEC's using a Mann-Whitney U-test. To validate our procedure, we applied our algorithm on the samples in gene expression data sets GSE10778 and GSE15464.

### Statistical analysis

Analysis of the gene signatures and array samples were done using BioConductor in R. Correlations were calculated with the Pearson correlation methods in SPSS 16.0 statistical software packages. Standard errors for Pearson correlation coefficients were estimated by the formula SE = (1-Rho∧2)/SQRT(n-1). Cox proportional regression models estimated survival hazard ratios with 95% confidence intervals. Meta-analysis was done using the MIX 2.0 software using a random effects model for relative risk and correlation coefficients.

## Results

### Generation of VEGF-A activation signature

**Figure 1:**
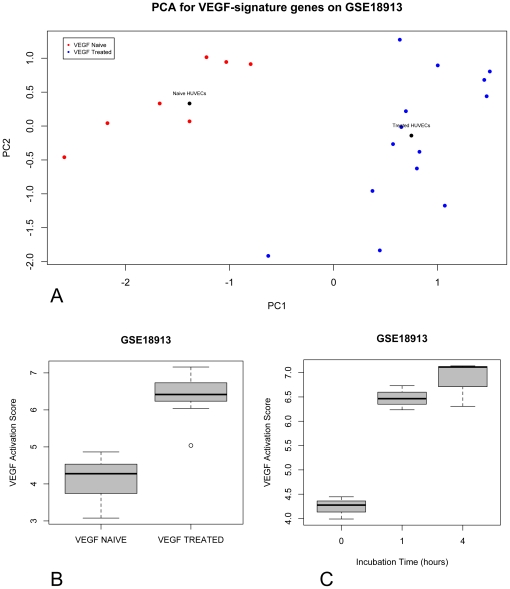
A VEGF-A signature was able to distinguish VEGF treated and naïve HUVEC's in the GSE18913 dataset. The 2D scatterplot of the principal component analysis showed that the centroïds had an observed Euclidean distance different from the expected Euclidean distance (p<0.0001). The first principal component of each sample is plotted along the X-axis, while the second principal component is plotted along the Y-axis. VEGF-A treated HUVEC samples are represented in blue and VEGF-A untreated samples are represented in red. Centroïds of both conditions are indicated by a black dot. (Panel A). After conversion to an activation score, the VEGF-A treated HUVEC's showed higher VEGF-A activation score in a time dependent relation (PANEL B AND C).

The VEGF-A signature described by Hu and colleagues [20] was derived from a matched analysis of primary tumours, lymph node metastases and distant metastases in breast cancer. A 13-gene profile, containing *VEGF-A*, discriminated between primary tumour samples and regional metastases on the one hand and distant metastases on the other hand. These 13 genes were analysed on publicly available gene expression data sets of naïve and VEGF-A treated HUVEC's. First, the discriminative power of the 13-gene signature to distinguish between VEGF-A treated and naïve HUVEC's was tested on gene expression data set GSE18913. Within this data set, only 10 out of 13 genes (*FABP5*, *UCHL1*, *PLOD*, *DDIT4*, *VEGF*, *ADM*, *ANGPTL4*, *NDRG1*, *NP* and *SLC16A3*) were reliably measured (high signal-to-noise ratio). Using these 10 genes in a principal component analysis (PCA) we were able to demonstrate a significant segregation of VEGF-A treated and naïve HUVEC's along the first principal component. Class label permutation analysis revealed that the observed Euclidean distance between the centroids of the VEGF-A treated and naïve HUVEC's on the 2D scatterplot representation of the PCA was significantly different from the expected Euclidean distance (Figure 1A; Observed Euclidean distance = 2.185, Expected Euclidean distance = 0.682, P<0.0001). Next, we transformed the VEGF-A signature into a VEGF-A activation probability score adopting the methodology described by Gatza and his colleagues [10]. Therefore, we used the regression coefficients that define the first principal component and multiplied these with the gene expression values of their corresponding genes. The products were summed and the resulting score was compared between VEGF-A treated and naïve HUVEC's using a Mann-Whitney U-test (Median VEGF-A treated HUVEC's: 6.416, Median naïve HUVEC's: 4.276, P<0.0001). The boxplot representation is provided in Figure 1B. In addition, we observed a strong correlation between the VEGF-A activation probability scores and the time of VEGF-A incubation of HUVEC's (Correlation coefficient = 0.762; P = 0.038). (Figure 1C)

**Figure 2:**
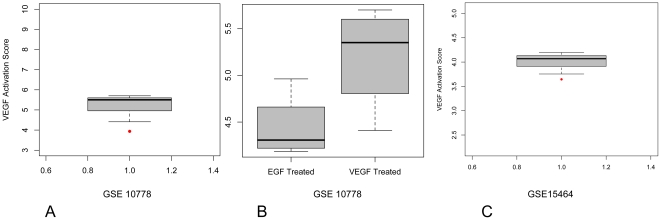
Validation of the VEGF-A activation score was performed in dataset GSE10778 (PANEL A) and GSE15464 (PANEL C). In both datasets, activation scores of VEGF-A treated HUVEC's (boxplots) were higher than the untreated condition (red dots). For GSE 10778, there was a higher activation score in VEGF-A treated cells, but not in EGF treated cells. (PANEL B).

In order to validate our VEGF-A activation probability score, we downloaded two more publicly available gene expression data sets (GSE10778 and GSE15464) on VEGF-A treated and naïve HUVEC's. In the first data set (GSE10778), the median VEGF-A activation probability score of the VEGF-A treated HUVEC's (N = 4; Score = 5.351) was significantly different from the score observed in the naïve condition (N = 1; Score = 3.937) (P = 0.0209). A boxplot representation is provided in Figure 2A. In addition, we observed a significant difference between the VEGF-A treated (N = 4; Score = 5.351) and EGF-treated HUVEC's (N = 4; Score = 4.307) (P = 0.0431), suggesting that the VEGF-A activation probability score is VEGF-A specific (Figure 2B). Finally, we performed the same analysis for data set GSE15464. In this case, we observed a trend towards a significant difference between the VEGF-A treated HUVEC's (N = 3; Score = 4.069) and the score observed in the naïve condition (N = 1; Score = 3.645) (P = 0.0892). Again, a boxplot representation is provided in Figure 2C. In these two data sets, we observed strong correlation coefficients between the VEGF-A incubation time and the VEGF-A activation probability score (respectively 0.503 and 0.692), however the p-values did not reach significance, probably due to the small sample sizes of the data sets.

### Application of pathway activation signatures

We applied the oncogenic pathways on the six datasets. These datasets together represent a total of N = 464 advanced serous papillary carcinomas. A summary of these 6 datasets is listed in Table 1. Since these are selected oncogenic pathways, it is plausible that many significant correlations were found between pathway activations and the 3 prognostic signatures (IGS, WHR and GGI). The β-Catenin pathway showed consistent and strong correlations. (Table 2) Since the six datasets were generated on different platforms with different methodologies, we estimated the overall effect of a pathway activation score by using a meta-analysis approach (Table 2). Similar meta-analysis of correlation coefficients showed that the BRCA, E2F1, EGFR, HER2, MYC, p53, p63 and PI3K showed steady correlations with the WHR, GGI and IGS. The RAS pathway and TGFβ pathway showed significant correlations with 2/3 prognostic signatures. Table 3 shows the overall correlation estimates, which were the most significant. While most pathway activation scores showed a positive correlation, the EGFR, HER2, p53 and TGFβ pathway showed a negative correlation.

**Table 1:** A summary of datasets that were used in the meta-analysis.

Dataset	N = 464	Platform	Normalisation	Clinical outcome	Uniform treatment	Advanced stage/serous papillary histology	Remarks
Québec 2006	20	Agilent Human 1A (v2)	Lowess	PFS	yes	yes	patients were selected either to be chemosensitive versus chemosensitive
North Carolina 2006	107	Affymetrix U133_plus2	GC-RMA	OS	yes	yes	
Melbourne 2008	165	Affymetrix U133_plus2	RMA	PFS	yes	yes	
Niigata 2010	110	Agilent Whole Human Genome Oligo Microarray	Upper quartile	PFS	yes	yes	
Boston A 2010	26	Affymetrix U133_plus2	RMA	PFS	yes	yes	all patients were BRCA1&2 mutation negative
Boston B 2010	36	Affymetrix U95_A2	RMA	PFS	yes	yes	dataset enriched for BRCA1&2 mutation negative patients/patients with no relevant family history of ovarian/breast cancer

**Table 2:** Table 2 shows the consistent correlations of the β-Catenin activation scores and WHR/IGS/GGI in each separate dataset (Québec, North Carolina, Melbourne, Niigata, Boston A and Boston B dataset).

Pearson Rho	WHR	IGS	GGI
Québec	0.65	0.62	0.67
	p = 3.4 E-4	p = 0.001	p = 2.0 E-4
North Carol	0.81	0.89	0.6
	p = 7.7 E-40	p = 9.9 E-59	p = 8.0 E-18
Melbourne	0.73	0.54	0.79
	p = 2.8 E-22	p = 6.9 E-11	p = 5.6 E-28
Niigata	0.77	0.73	0.79
	p = 2.4 E-19	1.0 E-22	p = 4.5 E-25
Boston A	0.83	0.48	0.87
	p = 1.2 E-7	p = 0.013	p = 5.5 E-9
Boston B	0.75	0.56	0.26
	p = 1.8 E-7	p = 3.7 E-4	p = 0.13
**Meta Analysis**	**0.73**	**0.62**	**0.79**
	**p<0.0001**	**p<0.0001**	**p<0.0001**

Overall Rho Coefficients were estimated by a meta-analysis approach using random models effects.

**Table 3:** Estimates of Pearson rho correlation coefficients after meta-analysis of six datasets between pathway activation scores and prognostic gene signatures: wound healing response signature (WHR)/Invasiveness gene signature IGS and Genomic grade Index (GGI).

Rho estimates	WHR	IGS	GGI
β-Catenin	0.73	0.62	0.79
	p<0.0001	p<0.0001	p<0.0001
BRCA	0.43	0.36	0.36
	p<0.0001	p<0.0001	p<0.0001
E2F1	0.51	0.42	0.54
	p<0.0001	p<0.0001	p<0.0001
EGFR	−0.52	−0.43	−0.42
	p<0.0001	p<0.0001	p<0.0001
HER2	−0.45	−0.5	−0.26
	p<0.0001	p<0.0001	p<0.0001
MYC	0.69	0.53	0.4
	p<0.0001	p<0.0001	p<0.0001
p53	−0.59	−0.42	−0.72
	p<0.0001	p<0.0001	p<0.0001
p63	0.46	0.29	0.36
	p<0.0001	p = 0.001	p<0.0001
PI3K	0.43	0.33	0.29
	p<0.0001	p<0.0001	p = 0.002
RAS	0.51	0.2	0.4
	p<0.0001	p = 0.017	p<0.0001
TGFβ	−0.23	−0.3	−0.13
	p = 0.0001	p<0.0001	p = 0.004

Most significant correlations are shown. (Threshold p-value adjusted for multiple testing = 0.0025).

### Survival Analysis

**Figure 3:**
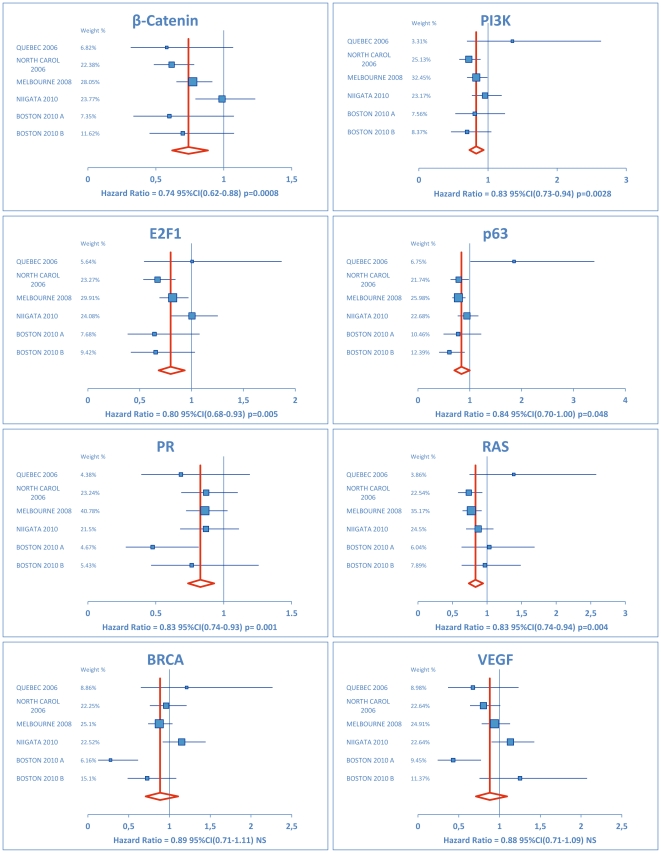
Forest plots of meta-analysis using a random effects model of the β-Catenin BRCA, E2F1, p63, PR, PI3K, RAS and VEGF pathway. The VEGF and BRCA signature was overall not significantly associated with clinical outcome. The other pathways showed significant association with survival after meta-analysis using 6 datasets (Québec, North Carolina, Melbourne, Niigata, Boston A, Boston B). Note the larger 95% Confidence intervals of the Québec dataset due to lower number of patients. Along the X-axis, hazard ratios are indicated by the centre of each square for each dataset. The meta-analysis used a weighted method (shown by the size of the squares/and the percentages indicated for each dataset) based upon confidence interval/number of patients. The 95% confidence interval for each hazard ratio is indicated by the width of the blue lines originating from the squares. The vertical red line shows the overall hazard ratio after meta-analysis, with the width of the diamond as the 95% confidence interval.

Next, we looked at clinical outcome and the degree of pathway activation. While some pathways were associated with survival outcome in one or more datasets, they showed no or opposite result in another dataset. To estimate the overall survival effect of a given pathway, a similar meta-analysis approach was performed to estimate the overall effect of pathway activation using a random effects model. After this analysis, the β-Catenin, E2F1, PR, p63 PI3K and RAS pathway activation showed a significant association with clinical outcome. Considering the overall effect by means of Hazard Ratios, the β-Catenin pathway showed the most prominent effect after meta-analysis (HR = 0.74; 95%CI [0.62–0.88]). The survival analysis showed that the higher the activation of the β-Catenin pathway, the better the outcome was. Also for PR, E2F1, RAS, PI3K and p63 increased activation of respective pathway was associated with more favourable survival. (Figure 3)

Because of these rather unexpected results, we calculated the activation scores of selected discovered pathways in other independent datasets as additional quality control to confirm whether the directions of the activation scores were certainly correct. For β-Catenin the activation scores were found to be higher (borderline significance, p = 0.06) in paediatric medulloblastoma with a *CNTBB1* mutation (N = 4) versus non-mutated medulloblastomas [21]. For the RAS activation score, *K-RAS* mutated colorectal cancers (N = 27) had a significant higher activation score (p = 0.03) than wild type cancers (N = 43) [22]. *PIK3CA*-mutated cancers (N = 14) had a higher -although non significant- PI3K activation score (p = 0.11) than non-mutated breast cancers (N = 29) [23]. The PR activation scores were higher (p = 0.06) in ER positive, PR positive breast cancers (N = 59) versus ER positive, PR negative breast cancers (N = 18) [24].

**Figure 4:**
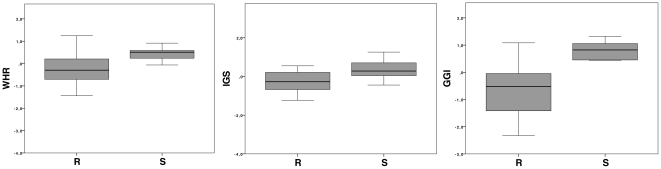
In the Québec dataset sensitive (S) patients showed a higher genomic grade index (GGI) compared to chemoresistant patients (R) (p = 0.002). Similarly chemosensitive patients showed a higher wound healing response score (p = 0.02) and a higher invasiveness gene signature score (IGS) (p = 0.06).

For the 3 prognostic signatures there was a tendency that a prognostic worse outcome predicted by IGS, WHR and GGI showed an unexpected higher probability of better clinical survival outcome. Further analysis in the Québec dataset showed that chemoresistant patients showed significant lower scores than chemosensitive patients and therefore may explain this finding. (WHR p = 0.02; GGI p = 0.002; IGS p = 0.06) (Figure 4)

## Discussion

Although VEGF-A inhibition has proven to be active in ovarian cancer, the VEGF-A activation signature we studied did not prove to be of any significant prognostic value in this meta-analysis. A number of considerations must be made: First, it may not be necessary that VEGF-A biology automatically would be of any prognostic value, even if VEGF-A inhibition has demonstrated to be effective [2], [3]. Secondly, although this signature was validated on gene expression data and was able to distinguish between VEGF-A treated versus untreated HUVEC's cells, it may lack the ability in clinical samples.

Earlier studies have shown that a signature derived from BRCA mutated vs sporadic disease was able to divide a population of sporadic epithelial ovarian cancers in tumours with a “BRCAness-like” and a ‘’non-BRCA-like” profile. These two groups had significant different clinical outcome in terms of overall and progression free survival. In the present meta-analysis this BRCA-profile was correlated with prognostic signatures, however it did not prove to be of significant prognostic/predictive value considering the total of 6 datasets. The difference in patient selection may partly account for this finding. Konstantinopoulos and colleagues selected patients enriched for sporadic cancers, which were predominantly BRCA1 and BRCA2 mutation negative [19]. Here, patients were selected for advanced stages, serous papillary histology and uniform chemotherapy regimen. Furthermore, we did not use a cut-off point to divide patients in BRCA-like and non-BRCA-like tumours. Since our objectives were to find biological associations, rather than to validate a specific biomarker in the perspective for possible clinical usage, we used the BRCA-activation score as a continuous variable in survival and correlation analysis.

Our initial analysis consisted of two datasets. The initial design was to use one dataset, as a discovery dataset while the other one would serve as a validation set. Since bioinformatical mislabelling errors/reproducibility issues have lead to withdrawal of papers of the same research group from which one dataset originated, we sought additional datasets to confirm our findings and render more power and reliability [25]–[27]. Furthermore, this research group and critical review by another research group have confirmed that the dataset that was used in the present meta-analysis was indeed correctly annotated [9], [28]. With the availability of more datasets, we noticed variation among pathway's association with survival outcome. We therefore used a meta-analysis approach to estimate the overall effect. The advantage is that several studies can be combined despite differences in platforms and methodologies. This overall effect estimation takes into account the number of patients of each separate dataset and confidence interval in the estimation of correlation coefficient of survival hazard ratios. The heterogeneity among datasets (e.g. different patient selection criteria) may partly explain some opposite findings. The Québec dataset is different from others because this specifically selected patients to study differential expression between chemosensitive versus chemoresistant tumours [17]. This dataset therefore may represent the extremities of this disease. Interestingly this dataset showed clearly that chemosensitive patients had tumours that were more likely to be of unfavourable outcome estimated by WHR/IGS/GGI. This contradictory finding may be explained by the finding that these three prognostic signatures are all primarily associated with increased proliferation [29]. It is known that chemosensitive tumours have higher tumour cell proliferation indexes in serous ovarian cancer [30], [31]. The estimated prognostic values in this survival analysis therefore seems strongly oppositely confounded by the predictive value for platinum/taxane-based chemotherapy.

**Figure 5:**
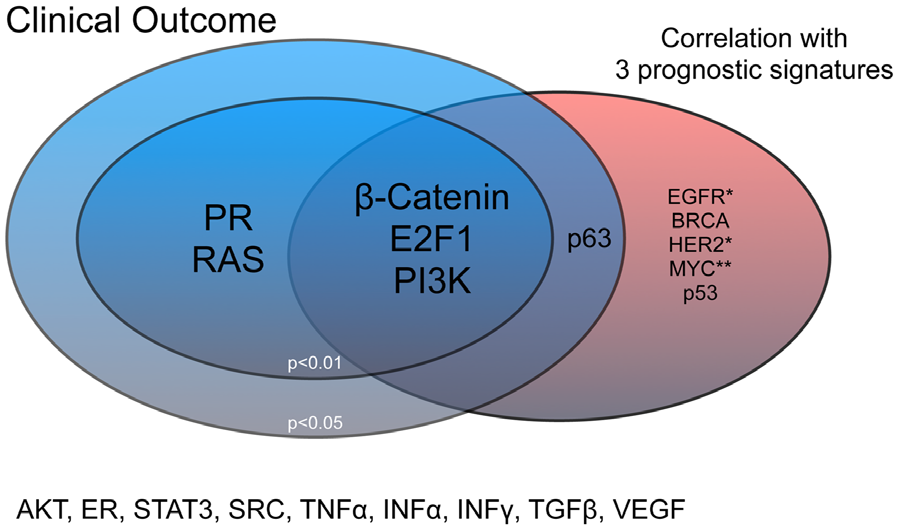
A Venn diagram is showing combined results of the meta-analysis: β-Catenin, E2F1, p63 and PI3K activation scores showed significant association with survival and were significantly correlated with all three prognostic signatures (WHR/IGS/GGI) after meta-analysis. PR and RAS activation scores were associated with clinical outcome, but did not consistently correlate with prognostic signatures. *Negative correlation coefficient **borderline significance with clinical outcome.

Despite the heterogeneity in datasets and confounding of predictive value versus prognostic value, the E2F1, β-Catenin and the PI3K activation scores showed overall association with survival outcome (p<0.01) and consistent significant correlations with three prognostic signatures. (Table 3+Figure 5)

The E2F1 pathway a critical role in proliferation and apoptosis. It has been shown that transcription factor E2F1 interacts with the p53 and PI3K pathway [32]–[34]. Its role in ovarian cancer has been unclear, as other research groups have found similar favourable survival with increased E2F1 pathway activation [32], while other findings have shown favourable survival with decreased *E2F1* gene expression by RT-PCR [33], [34]. It must be remarked that the latter study included an overrepresentation of patients with clear cell carcinomas (42.9%) and may be less informative here.

The β-Catenin protein is a multifunctional protein. It was originally discovered as a protein that is associated with the cytoplasmatic region of E-cadherin. E-cadherin is a transmembrane protein that is involved in cell-cell contact and cell's adhesive functions. Furthermore, β-Catenin is involved in Wnt signalling as a nuclear transcription factor and is believed to play a role in cancer stem cells [35]. Loss of its membranous function or a higher nuclear presence has been linked with poor survival in several studies in ovarian cancer based upon immunohistochemical studies [36]–[40]. In addition, a correlation of β-Catenin protein expression has been described with tumour grade and Ki-67 expression [39], [40]. Present results are thus confirmative of earlier findings that β-Catenin is associated with survival outcome. The consideration must be made whether this effect is not attributed to its predictive value to platinum-taxane chemotherapy rather than its prognostic value. In present study, β-Catenin had strong and consistent correlation with IGS/WHR/GGI. Although these signatures were constructed based upon different oncogenic biological processes (wound healing, stem cell phenotype, grade), their major common force has been proven to be cell proliferation [29]. The observation that chemosensitive patients in present analysis showed significantly higher values of GGI, WHR and IGS renders credibility to this statement.

Similarly, the unexpected findings that increased activation of PI3K-, and RAS- pathways are more favourable for survival may be explained by their predictive value for chemotherapy. This hypothetically may have clinical consequences. Several compounds target the PI3K pathway or downstream effectors (e.g. mTOR) and are under early clinical development in epithelial ovarian cancer. Other compounds have inhibitory effects on the RAS pathway, e.g lonafarnib (a farnesyltransferase inhibitor). Recent findings of a randomised phase II trial (IGCS meeting 2010, W. Meier et al.) showed that the concomitant addition to standard chemotherapy (first line) and 6-month continuation of lonafarnib in primary epithelial ovarian cancer stage IIB-IV (n = 105) resulted in borderline poorer outcome for the experimental- lonafarnib arm (overall survival HR = 0.62 95CI%(0.36–1.06) p = 0.08) or even resulted in significant unexpected worse outcome (p = 0.01) in the experimental stratum of patients with suboptimal debulking. This finding may be relevant in the context of our results. Since increased activation of pathways as RAS and PI3K have been found to be favourable for survival outcome, the question should be asked whether inhibition of one of these pathways concomitant with chemotherapy is desirable. These pathways are driving forces of proliferation, which is an important factor in the efficacy of standard chemotherapeutics. We hypothesize that inhibition of these pathways may therefore also negatively affect the efficacy of these chemotherapies and theoretically induce chemoresistance. This would possibly be an explanation for the recent unexpected findings of lonafarnib in ovarian cancer. Hence, we theorize that these agents may have their potential in ovarian cancer in a sequential adjuvant setting rather than its concomitant combination with chemotherapy.

The PR pathway did not show any relevant association with IGS or GGI. It did show high significant association with survival outcome and WHR. Other immunohistochemical studies have shown that the PR protein expression has predictive of prognostic value, more than the expression of ER [41]–[44], Since PR expression is a downstream target of the ER pathway, this finding may indicate that an active ER pathway, rather than the expression of ER by itself may be of importance. Anti-hormonal therapies have shown anti-tumoural activity in relapsed/refractory ovarian cancer in phase II studies [45]–[49]. Biomarker studies have shown that increasing ER expression was associated with increasing CA125 response rate [47]. We suggest that further studies are needed to study if PR expression may add value as a suitable biomarker to select patients for anti-hormonal therapy in ovarian cancer.

To conclude, oncogenic pathway profiling of advanced serous ovarian tumours revealed that it is difficult to estimate the true prognostic value of a pathway since there seems confounding of predictive factors. Despite these biases, with a meta-analysis approach of 6 independent datasets generated on different micro-array platforms, we found that a PR and a RAS activation score was associated with clinical outcome. Activation scores for β-Catenin, p63, E2F1 and PI3K were also associated with survival and were consistently correlated with three prognostic gene signatures. Further studies are needed to elucidate whether these pathways may help in designing targeted therapies treatment strategies.
